# Mr. Tripe in Reply to Mr. Smith's Observations Respecting the Frequency of Calculous Disorders in Great Britain

**Published:** 1821-10

**Authors:** 


### Mr. Tripe *in Reply to* Mr. Smith's *Observations respecting the Frequency of Calculous Disorders in Great Britain.*

IN answer to the call upon me, contained in Mr. Smith's reply to my observations upon his Statistic Inquiry into the Frequency of Stone in the Bladder, I now beg leave to state, that, up to the latter end of 1816, I had cut, in my practice, nine cases. I have also made the most careful inquiry upon the subject, and have ascertained that there have been cut, in these towns and their vicinity, by other surgeons, during the twenty years previous to and ending in 1816, twenty-five cases; but the most of them occurred within the latter half of that period. Since 1816, there have been seven operations for stone, besides the four which I performed in my practice in the same interval. I have not extended my inquiries further back than 1796, because it is well known that before that date such operations were rarely, or never, performed in this part of the country.

I am quite certain, from the very candid and liberal manner with which Mr. Smith has proposed to treat this subject, that it was not his intention, by any thing which he has written in his reply, to leave the least impression upon the mind of any individual, which could be unfavourable to my intentions; but, as one part of his paper contains sentences written in such a connexion as are liable to lead his readers into a suspicion that, in my former statement, I have not been accurate, and therefore not strictly just towards Mr. Baines, I trust that I shall stand excused for having any anxiety to prevent the possibility of this effect being produced.

I would beg leave to observe to Mr. Smith, that Mr. Baines's report of the number of cases which had been operated upon at the Exeter Hospital, and published in his Inquiry, does not end in 1816: the words of the report are, "between the years 1806 and 1816, eighteen cases; between 1811 and 1818, nine cases."

The cases I have now enumerated from this neighbourhood, for the purpose of enabling Mr. Smith to rectify the error which he has been unconsciously led into, in the publication of his Inquiry, will, at the same time, prove that the numbers which I before published in my Observations were not overrated.

The object with which I wrote these observations was to exhibit the mistake which Mr. Baines committed, when he assigned to the Institution, of which he is a surgeon, the merit of performing, with very few exceptions, all the stone-operations for the county of Devon, &c.; and, as well, to prove that the deduction made by Mr. Smith from his Inquiry, as far as it regarded the frequency of the disease in this county, was the reverse of what it should have been: and I stated the number of operations which had been performed in these towns during the last ten years, (twenty-six,) to illustrate the fact, by comparing it with the last ten years of the hospital report; at least, as far as we were enabled to judge of it, from the manner in which that report is drawn up.

The subjects of those operations were mostly from the poorer classes of society, residing in crowded and unwholesome apartments, whose meals consist principally of vegetables, occasionally varied by fish, and these not always in sufficient quantity: it is hardly necessary to add, that their drink is water.

The following interesting fact, communicated to me, in the course of my inquiries upon this subject, by an intelligent medical friend who practises in these towns, is deserving of being recorded.

A few years since, a man, aged about twenty-five, applied to him for his assistance; and, on examination of his urethra, a stricture was found at about three inches from the orifice, which was hard and cartilaginous in its feel. No instrument could be made to pass this obstruction; he therefore had recourse to the bougie, armed with argent. nitratum, which succeeded in forcing the passage at this part, and with but little irritation or trouble, more than is commonly experienced by this mode of treatment. Another stricture, of the same character as the former, was found at five inches; and the application of the caustic had made a considerable impression upon it, when symptoms of great irritation intervened, and arrested its further progress. Inflammation, and a circumscribed tumor, formed in the perineum, which eventually burst, and discharged a quantity of matter, succeeded by a constant flow of urine. The opening made in the perineum soon became very considerable, from the sloughs which were thrown off. On examining the sinus, with a view to ulterior treatment, a calculus, larger than a sparrow's egg, which had ulcerated its way through the coats of the bladder close to the prostate, was discovered lying near that gland: it was immediately extracted by the common dressing forceps. The sloughing, however, increased, and, from the incontrollable and constant extravasation of the urine which followed, it was extended to the surrounding parts; which cause of irritation, together with extensive excoriations, became too much for his previous shattered state of health, and it soon destroyed him.

I have met with two additional cases of calculus since I wrote my former observations: one, a man aged seventy-five, the other sixty-five. The latter having been subjected to the operation, and his case being of some interest, I shall take the liberty of briefly stating it.

George Harvy, aged sixty-five, a labouring man, and of meagre habit, having been afflicted with an urinary disease during the last twenty-five years, applied to me to ascertain if he had a stone in his bladder, and, if he had, to perform the necessary operation.

The introduction of the sound into the bladder was opposed by a calculus, which, from its extent of surface, inclined me to believe that it was more than of ordinary magnitude. The cavity of the bladder was diminished, and its coats apparently thickened and diseased. For the last five years he had been unable to retain any quantity of urine, being necessitated constantly to discharge it; and it exhibited a copious sediment of puriform mucous.

It was explained to him, that these unfavourable circumstances threw more than usual doubts in the way of his recovery from an operation; and therefore, although I would not recommend it, yet, as it afforded the only chance of prolonging his life beyond a very limited period, if he continued desirous to have it performed, I should feel it my duty to accede to his wishes. Having resolved upon the operation, the difficulty of contending with a large stone naturally occupied our consideration. The operation above the pubis could not be selected, because of the contracted state of the bladder; and, therefore, the only resource was to be provided with the means of breaking the stone, if it should be found necessary. But I have to regret, that an instrument well adapted for this purpose still remains a desideratum. In answer to my application to a respectable surgeon's instrument maker in London, I was informed that the most improved instrument for breaking the stone had very lately so completely failed in the hands of an expert lithotomist, that he did not feel warranted in sending it.

I performed the operation in the presence of many of the most respectable professional characters. Both the external and internal incisions were completed with the common scalpel, and formed an ample and easy passage to the cavity of the bladder. Although every precaution was used to insure a retention of as much water as possible, yet only a small quantity trickled over the hand, upon the prostate and neck of the bladder being divided. The calculus immediately presented, and the staff, whose extremity could only be introduced just within the neck of the bladder, was withdrawn.

The cavity of the bladder appeared quite filled with calculous matter. The forceps were applied, and every means adopted to carry on the blades between the parietes of the bladder and the stone sufficiently to grasp the body of it; there did not appear for this purpose an adequate space. Enough, however, of the stone was embraced by the forceps to allow of a considerable force being used, and after several efforts it was fortunately broken; when I was enabled, by the repeated introduction of the forceps and the scoop, and by washing, to clear the bladder of its contents. This part of the operation was the more tedious, as it became necessary, during the process, occasionally to detach, in different parts of the bladder, but particularly in the anterior superior part, calculous masses, which were lodged and retained in the furrows and cavities which appeared to be formed in the diseased internal surface of the bladder.

The patient, after the operation, and until the third day, was free from any unfavourable symptom. His countenance was then sunken, and his pulse was weak and irritable; he complained of pain in the left groin, and occasionally in the abdomen, but not severe; his belly was tense, and he had vomiting. His bowels had been moved by a purgative, which he had taken the previous night. Ordered twelve leeches to the lower part of the belly, and to be subsequently fomented; a purgative enema to be injected; and a grain of calomel, with half a grain of opium, in a pill, every two hours. He gradually sunk without any severe pains, and expired at nine P. M.

An opportunity being afforded, the next morning the body was examined. The vessels of the peritoneum covering the intestines, and where it is reflected over the bladder, were injected, in very small distinct patches, in different parts of it: no other evidence of inflammation within the abdomen could be discovered. The bladder was removed from its situation, and laid open. The internal surface of it was formed into deep rugæ and hollows, was of a chocolate colour of different shades, and exhibited in some places spots of ulceration. The substance of the bladder was thickened and hard throughout; but the thickening was greatest, about an inch and half in width, in the direction of a line drawn perpendicularly from the prostate through the fundus, and descending to the anterior and inferior part, by which the cavity of the bladder was most contracted through the centre, and its fundus divided into two sacculi.

The prostate was healthy; the structure of the kidneys was also natural, excepting a grain or two of the earthy phosphate found in the infundibulum of the left, and some enlargement of their pelves. The ureters were enlarged, but not so considerably as the state of the bladder would have warranted the anticipation of.

The calculus is composed of a lithic nucleus, shaped like a broad bean, but larger; its coating, strata of phosphate of lime, of different thickness and density; its size, when entire, exceeded a duck's egg. The fractured parts of the calculus, when brought together, exhibit a depression on its posterior surface, which probably was formed by its contiguity to the ridge on the posterior part of the bladder; and there also appear projections at its upper and lateral parts, forming shoulders which correspond with the hollows at the fundus. Besides this, a quantity of calculous matter, coloured like the exterior of the stone, and of the consistence of mortar, lay in the pouches at the fundus of the bladder, filling up the intermediate space between it and the stone.

The superior advantage of the scalpel in completing the opening into the bladder, inasmuch as its use depends less upon mechanical contrivance, and is therefore more under the control of the operator, by which he is enabled to effect the necessary division of parts, without incurring those risks which more or less, according to the particular skill of the surgeon, must always attend the gorget and lithotome cachi, is, I believe, now very generally admitted; as well as the benefit of being enabled to proportion the opening through the prostate to the size of the calculus.

But the case before us illustrates still more strongly the propriety of giving to the scalpel the preference. If the lithotome caché or the gorget had been selected for making the incision into the bladder, the attempt must have failed, and, if persisted in, might have been productive of much mischief.

The difficulty in this case did not centre in the size of the stone, but in its connexion with the diminished cavity and unyielding state of the bladder.

As far as I am acquainted with the principles upon which instruments must act to break a stone *in situ*, they can only be applied in cases when the disproportion is between the size of the stone and the opening which can be made with safety in the neck of the bladder: that is, there must be sufficient space in the bladder to admit of the instrument being so applied upon the stone as to effect a resistance from behind, which shall be equal to the power that is exerted upon its surface, in order to crush it. I am quite certain that no such means could have been employed in this case; and, if the calculus had been formed of more solid structure, there is no way by which the bladder could have been relieved from it, which would have been at all consistent with a chance of the patient's recovery.

*Plymouth Dock; August 16th,* 1821.

---

